# Family Outbreak of Shiga Toxin–producing *Escherichia coli* O123:H–, France, 2009

**DOI:** 10.3201/eid1609.100472

**Published:** 2010-09

**Authors:** Lisa A. King, Ingrid Filliol-Toutain, Patricia Mariani-Kurkidjian, Véronique Vaillant, Christine Vernozy-Rozand, Sarah Ganet, Nathalie Pihier, Patrick Niaudet, Henriette de Valk

**Affiliations:** Author affiliations: Institut de Veille Sanitaire, Saint Maurice, France (L.A. King, V. Vaillant, H. de Valk);; Institut Pasteur, Paris, France (I. Filliol-Toutain);; Hôpital Robert Debré, Paris (P. Mariani-Kurhidjian);; VetAgro Sup Campus Vétérinaire de Lyon, Marcy l’Etoile, France (C. Vernozy-Roxand, S. Gantet);; Direction Générale de l’Alimentation, Paris (N. Pihier);; Hôpital Necker Enfants Malades, Paris (P. Niaudet)

**Keywords:** Shiga toxin–producing Escherichia coli O123:H–, Escherichia coli, disease outbreaks, foodborne diseases, zoonoses, ground beef, bacteria, France, letter

**To the Editor:** Shiga toxin–producing *Escherichia coli* (STEC) is a major cause of foodborne disease in industrialized countries. We present results of the investigation of a family outbreak in France caused by a rare STEC serotype.

Surveillance of STEC infections in France since 1996 has been based on national surveillance of STEC-related pediatric hemolytic uremic syndrome (HUS) (*1*). On February 11, 2009, two cases of diarrhea were reported to a surveillance coordinator: 1 in a child with HUS and the other in that child’s sibling.

The 2 siblings, 2 and 6 years of age, had diarrhea beginning on February 4 and 5, 2009. Bloody diarrhea developed in the younger child, and HUS was diagnosed on February 9. The older child had nonbloody diarrhea for 3 days and abdominal pain. Questioning of the patients’ parents identified no recent history of travel, contact with farm animals, or outdoor bathing. A food history indicated that the 2 patients had shared an undercooked ground beef burger 4–5 days before symptom onset. The patients’ parents also ate burgers from the same package (box); they did not report any gastrointestinal symptoms.

Fecal specimens of the patients were tested for STEC by direct PCR for STEC genes (*stx*); after which culture and identification of *stx1*, *stx2, eae*, and *ehxA* (*hlyA*) virulence genes; and serotyping with a panel of 22 serum samples were conducted as described (*1**,**2*). Molecular serotyping was subsequently conducted on nonagglutinating strains by using the *rfb*–restriction fragment length polymorphism technique for O antigen (*3*) and sequencing of the *fliC* gene for H antigen (*4*).

A trace-back investigation was conducted for the implicated beef burgers, which were obtained from a box of 10, frozen, 100-g ground beef burgers purchased in late January 2009. The remaining beef burger in the box from which the patients had eaten a beef burger was obtained from the family’s freezer for microbiologic testing. Stored production samples from the implicated batch underwent microbiologic testing.

After broth enrichment, ground beef samples were tested by PCR for *stx* and *eae* virulence genes and O antigens of serotypes O157, O26, O145, O103, and O111 (*2**,**5**,**6*). Subsequently, strains isolated from *stx*-positive and *eae*-positive enrichment broths were biochemically tested and underwent serotyping and PCR identification of virulence genes. Genetic relatedness of clinical and ground beef STEC strains was studied by using pulsed-field gel electrophoresis with *Xba*l as described (*7*).

A nonmotile strain of STEC *stx2 eae ehxA*, which was not serotypeable by the panel of 22 serum samples, was identified in fecal samples from patients and in the remaining ground beef. Molecular serotyping of clinical isolates and an isolate from the beef identified a strain of STEC O123:H2. Analysis by pulsed-field gel electrophoresis indicated that the clinical and meat isolates were genetically related (Figure). The level of STEC contamination in the meat was 30–40 CFU/g. All stored meat production samples tested were negative for STEC.

**Figure:**
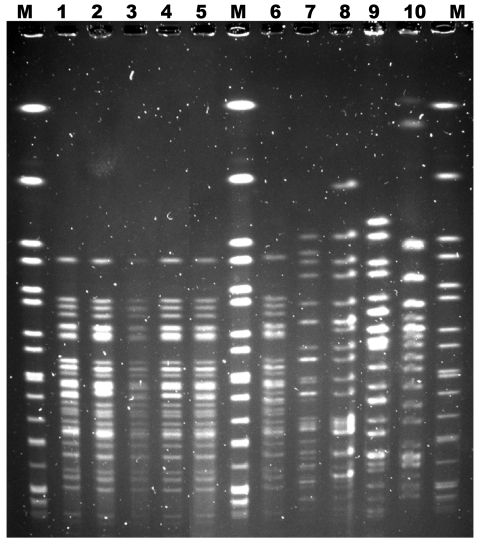
Representative *Xba*I pulsed-field gel electrophoresis patterns of Shiga toxin–producing *Escherichia coli* (STEC) O123:H– strains isolated from patient fecal samples and strains isolated from ground beef obtained from patients’ home, France, 2009. Lanes M, *Xba*I-digested genomic DNA from *Salmonella enterica* serovar Braenderup H9812 used as molecular mass markers; lane 1, Shiga toxin–producing STEC O123:H– isolated from patient with hemolytic uremic syndrome; lane 2, STEC O123:H– isolated from patient with diarrhea; lanes 3–6, STEC O123:H– isolated from ground beef from patients’ home; lanes 7 and 8, STEC O123 reference strains; lane 9, STEC 0111 isolate not related to the strains of the patients; lane 10, STEC 0157:H7 isolate not related to the strains of the patients.

A clinical strain and a ground beef STEC strain were sent to the World Health Organization Collaborating Centre for Reference and Research on *Escherichia* and *Klebsiella* in Copenhagen, Denmark, in December 2009 for analysis. The clinical strain was confirmed as STEC O123:H–, and the meat strain was confirmed as a nonmotile STEC rough type by serum agglutination. Both strains had virulence genes *stx2a*, *eae*, and *ehxA* (F. Scheutz, pers. comm.).

We identified a family outbreak of STEC O123:H– *stx2a*, *eae ehxA* infections associated with ingestion of undercooked ground beef. No similar cases of STEC infection were identified by active case finding. This serotype is rarely described as a cause of human clinical infection. No human isolate of serotype O123:H– is recorded in the database of the World Health Organization Collaborating Centre for Reference and Research on *Escherichia* and *Klebsiella* (F. Scheutz, pers. comm.).

Two strains of STEC O123:H– *stx2d* were isolated from asymptomatic persons in Germany during 1996–2000 (*8*). A study in Australia in 2003 reported using a strain of O123:H– *stx1 stx2 ehxA* from Switzerland that had been isolated from a person with diarrhea (*9*).

We report foodborne transmission of STEC O123:H– that resulted in a cluster of clinical cases of infection. Eating ground beef is a well-established mode of STEC transmission, particularly for serotype O157:H7. STEC serotype O123:H– has been isolated from feces of healthy lambs and sheep in Spain (*10*) and in southwestern Australia (*9*) and is considered to be among the predominant ovine STEC serotypes in these countries.

This family outbreak shows that STEC serotype O123:H–, albeit rarely described as causing human illness, can cause severe human infection. This serotype can also cause clusters of STEC infections and be transmitted by ingestion of undercooked ground beef.
